# Factors Associated with Decisional Regret After Shared Decision Making for Patients Undergoing Total Knee Arthroplasty

**DOI:** 10.3390/healthcare13131597

**Published:** 2025-07-03

**Authors:** Yu-Chieh Lo, Yu-Pin Chen, Hui En Lin, Wei-Chun Chang, Wei-Pin Ho, Jia-Pei Jang, Yi-Jie Kuo

**Affiliations:** 1 Department of Orthopedics, Taichung Veterans General Hospital, Taichung 407219, Taiwan; b101106096@tmu.edu.tw; 2 Department of Orthopedics, Wan Fang Hospital, Taipei Medical University, Taipei 11031, Taiwan; 99231@w.tmu.edu.tw (Y.-P.C.); b101102088@tmu.edu.tw (H.E.L.); 99292@w.tmu.edu.tw (W.-C.C.); weipinho@w.tmu.edu.tw (W.-P.H.); 3 Department of Orthopedics, School of Medicine, College of Medicine, Taipei Medical University, Taipei 11031, Taiwan; 4 Department of Nursing, Wan Fang Hospital, Taipei Medical University, Taipei 11031, Taiwan; 93175@w.tmu.edu.tw

**Keywords:** total knee arthroplasty, decisional regret, shared decision making, post-operative pain, TKA

## Abstract

**Introduction:** Total knee arthroplasty (TKA) is a treatment for knee pain, but some patients are not satisfied with their outcomes. Utilizing shared decision making (SDM) can lead to better decisions, satisfaction, and fewer regrets. However, healthcare professionals have little knowledge of risk factors for regret. The aim of this study is to evaluate decisional regret using the Decision Regret Scale (DRS) after primary TKA among patients who engaged in SDM. **Method:** A total of 118 patients who underwent TKA surgery between March 2020 and May 2022 participated in this study, and they were able to reflect on their outcomes. The primary outcome was decisional regret assessed using the DRS, and the secondary outcome was post-operative pain at a three-month follow-up, measured using the Lequesne Index. **Result:** The study found that 49% of the patients reported no regret, 25% reported mild regret, and 26% reported moderate-to-severe regret. There was a significant correlation between greater levels of decision regret and a higher three-month Lequesne Index. Post-operative pain and post-operative mobility status and the range of motion of the knee joint were also strongly correlated. **Conclusion:** The study found that more than half of the patients undergoing primary TKAs experienced regret even following SDM counseling. Regret levels were associated with higher post-operative pain and poorer mobility. This underscores the importance of informing patients about potential adverse effects of TKA to manage their expectations and reduce regret in future SDM interviews. **Practice implications:** This study incorporated patient perspectives through their direct engagement in the SDM process prior to surgery. Patients participated in the design of the SDM framework, which included educational pamphlets and structured interviews to assess their values and preferences. Their involvement ensured that the SDM procedure was tailored to patient-centered outcomes. Furthermore, the follow-up assessments were conducted with patients to evaluate decisional regret and post-operative outcomes, providing valuable insights into the effectiveness of the SDM process. By actively participating in the research through decision making and outcome reflection, the patients contributed to the understanding of factors influencing decisional regret after undergoing TKA.

## 1. Introduction

A total knee arthroplasty (TKA) is one of the most effective and cost-effective treatment options for patients with osteoarthritis or osteoporosis who suffer from pain and stiffness in their knees [1]. In general, satisfaction rates following TKA vary, with studies reporting rates ranging from 65% to 100%, while dissatisfaction rates range from 8% to 20% [1,2,3,4]. Common factors influencing satisfaction and dissatisfaction rates following TKA include higher pain and function scores in the Oxford Knee Score [1,5], passive knee flexion [6], anxiety and depression [7,8], and female gender [3]. Decision regret (DR) has been used in many clinical areas, ranging from cancer services to diabetes, depression, treatment of lower back pain, and drugs for rheumatoid arthritis, and it is increasingly being seen as an important and useful patient-reported outcome measure (PROM) [9]. Given that regret is a common emotional experience, it is unclear how much regret occurs after health decisions are made, and what factors increase the probability of regretting these choices [10]. Currently, little is known about the risk factors that predict patients’ regret of their decisions, so healthcare professionals are not well equipped to anticipate or prevent it [11].

When a shared decision-making process (SDM) is utilized during the course of a clinical encounter, it allows patients to make treatment decisions together with their physicians [12]. Research has shown that patients who use shared decision aids are better informed, perceive risk more accurately, are more satisfied with the decision-making process, and have fewer regrets and conflicts as a result [13]. To date, numerous studies have employed shared decision making (SDM) to investigate the expectations of patients undergoing TKA. Patients who engage in SDM for TKA typically hold positive expectations regarding improved function and quality of life. However, common concerns include worries about the duration of their recovery, potential complications, anesthesia, their level of trust in the surgeon, financial implications, and their overall health [14]. Regarding post-operative satisfaction and regret levels among patients who underwent TKA with SDM, a notable subset experiences decision regret, with varying degrees reported [9]. Furthermore, research supports that SDM can lead to enhanced patient-reported outcome measures (PROMs) and patient satisfaction following TKA [13]. Additionally, studies have underscored that women, potentially due to a lesser understanding of osteoarthritis and TKA, demonstrate lower satisfaction with the decision-making process, especially concerning the anticipated recovery period post-surgery [15]. This factor may influence their willingness to undergo TKA.

To gain a more comprehensive understanding of the factors that may influence patient DR after TKA, our study aims to utilize SDM as a tool to assess post-TKA patient regret levels, explore reasons for regret, and identify specific demographics prone to decision regret.

## 2. Materials and Methods

This study uses a prospective cohort of 118 individuals recruited from single care centers in Taipei, Taiwan between March 2020 and May 2022, who made a shared decision to undergo TKA. Inclusion criteria were patients who had undergone primary TKA and patients engaged in a shared decision-making process prior to surgery. The excluded criteria were patients who had a revision of TKA or any condition other than osteoarthritis, and patients who were unable to give informed consent. All 118 individuals who were included in the study completed a follow-up assessment at three months. The primary outcome was decisional regret assessed using the DRS, and the secondary outcome was post-operative pain at three-month follow-up, measured using the Lequesne Index. This dual-indicator approach was chosen to differentiate emotional outcomes (regret) from physical recovery metrics (pain and function), thereby allowing exploration of how objective post-operative states contribute to subjective regret. The decision regret score reflects patients’ psychological appraisal of the decision process and outcome, while the Lequesne Index provides a validated measure of functional limitation, which is a common source of dissatisfaction after TKA.

The SDM procedure involved providing educational pamphlets and informing patients of the pros and cons of various options during their first outpatient visit. These pamphlets were adapted from AHRQ’s SHARE Approach toolkit and designed to help patients understand the indications, benefits, risks, recovery timelines, and functional expectations associated with total knee arthroplasty. In the second outpatient visit, healthcare providers discussed and confirmed the surgical method with the patient. This consultation focused on eliciting the patient’s concerns, clarifying their functional goals, and addressing potential sources of decisional conflict, such as fear of complications, caregiving responsibilities, or prior negative experiences. As part of the final outpatient consultation before surgery, a 20 min SDM interview was conducted with the patient and his or her family. The implementation of SDM was made possible by the use of a comprehensive tool developed by the Agency for Healthcare Research and Quality (AHRQ) [16]. By helping healthcare providers to incorporate SDM strategies into routine patient encounters, this SHARE (Seek, Help, Assess, Reach, Evaluate) approach was established with patient-centered outcomes in mind and outlines the following five essential steps: (1) seeking the patient’s participation; (2) helping the patient explore and compare treatment options in plain language; (3) assessing the patient’s values and preferences related to pain, function, and quality of life; (4) reaching a joint decision with the patient and their family; and (5) evaluating the patient’s confidence in their decision, with attention given to their remaining concerns or uncertainties. All providers involved in the SDM process attended a two-hour workshop based on AHRQ’s SHARE training curriculum, which emphasized patient-centered communication skills, use of teach-back to confirm their understanding, and strategies to support patients through uncertainty or emotional conflict.

Decision regret (DR) was measured using the Decision Regret Scale (DRS), a validated and widely used instrument developed by Brehaut et al. to assess regret following healthcare decisions from the patient’s perspective [17]. The DRS was originally designed for use across a range of medical contexts and has demonstrated good internal consistency (Cronbach’s alpha > 0.80) and construct validity. The DRS comprises five statements: (Q1) “It was the right decision”, (Q2) “I regret the decision that was made”, (Q3) “I would make the same decision if I had to do it again”, (Q4) “The decision did me a lot of harm”, and (Q5) “The decision was a wise one”. Patients rate each item on a 5-point Likert scale ranging from 1 (strongly agree) to 5 (strongly disagree). Items 2 and 4 are reverse-scored. The total score is linearly transformed to a 0–100 scale, with higher scores indicating greater levels of regret. As per established thresholds, a DRS score of 0 indicates no regret, 1–25 represents mild regret, and >26 signifies moderate-to-severe regret (Supplementary Table S1) [18].

The demographics of the patients (sex, age, BMI, CCI score, diabetes history, smoking status, level of education, operating site location, and previous history of contralateral TKA), their preoperative characteristics (ASA, outpatient visits within the 12 months prior to surgery, preoperative Lequesne Index, and duration of symptoms present prior to surgery), their perioperative characteristics (operation time and blood loss), and their post-operative characteristics at 3 months (DRS, mobility status, range of motion of knee joint, post-operative Lequesne Index, and hospitalization duration) were obtained from a hospital-maintained database. We measured the Lequesne Index before and 3 months after surgery at two different times. DRS was evaluated in patients 3 months after TKA. The Lequesne Index is a validated scale assessing pain and functional impairment in patients with knee osteoarthritis, consisting of 10 items across three domains: pain, maximum walking distance, and activities of daily living.

The statistical analysis was performed using SPSS version 26. For the primary outcome, we utilized Chi-square tests for categorical variables and ANOVA tests for non-parametric continuous variables. As for the secondary outcome, we employed Student t-tests for categorical variables and univariate linear regression for continuous variables. Furthermore, a multivariate regression analysis was utilized to identify significant variables and factors associated with post-operative Lequesne Index, while controlling for preoperative Lequesne Index as a confounding factor. The threshold for statistical significance was set at *p* < 0.05.

## 3. Result

**Figure 1:**
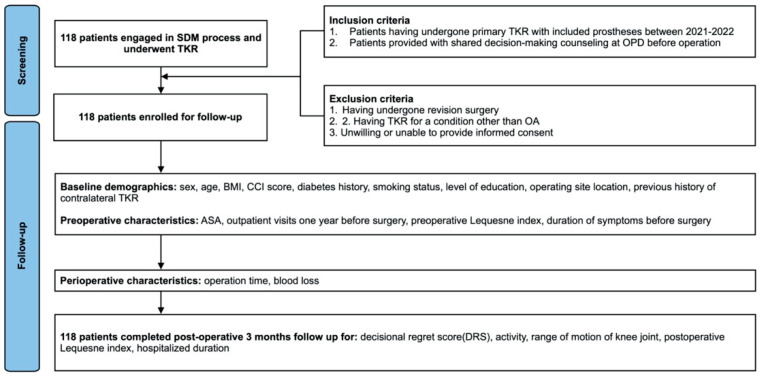
Flow diagram of study selection.

The study selection flow diagram is presented in Figure 1. Following screening and application of inclusion and exclusion criteria, 118 patients completed the 3-month follow-up period. The characteristics of the patients who underwent TKA are shown in Table 1. Of the 118 patients, 87 (73.7%) were female. The mean age was 72.31 years (SD 7.83), and the mean BMI was 28.16 kg/m^2^ (with an SD of 4.82). The average decision regret score among the total 118 patients was 18.22 (with an SD of 24.69). The average post-operative Lequesne Index was 5.02 (with an SD of 4.68).

The results for the three categories of decision regret, including no regret, mild regret, and moderate-to-severe regret, are shown in Table 2. Three months after surgery, out of all 118 patients, 58 (49.1%) patients had no regrets, 29 (24.6%) had mild and moderate-to-severe regret, and 31 (26.3%) had severe regret. The average post-operative Lequesne Index was 3.64 in the group without regrets, 4.59 in the group who had mild regret, and 8 in the group who had moderate-to-severe regret. The sociodemographic and preoperative/perioperative variables and factors such as sex, age, BMI, diabetes history, level of education, and previous history of contralateral TKA, among others, did not have a significant impact on the DRS scores at 3 months after TKA or affect the patients’ decision regret following TKA. However, post-operative mobility status (*p* = 0.005) and post-operative three-month Lequesne Index (*p* = 0.000) were found to be significant predictors of decision regret, indicating that patients who reported higher levels of post-operative mobility status and had lower Lequesne Index scores were less likely to experience decision regret after TKA.

According to the results presented in Table 3, the post-operative Lequesne Index was significantly associated with diabetes (*p* = 0.013), post-operative mobility status (*p* = 0.000), and the range of motion of the knee joint (*p* = 0.002). However, the multivariate linear regression analysis, with the results displayed in Table 4, showed that only post-operative mobility status and the range of motion of the knee joint reach statistical significance at a significance level of *p* < 0.05. This indicates that post-operative mobility status and the range of motion of the knee joint are significant predictors of higher post-operative Lequesne Index scores.

## 4. Discussion

The results of our study show that, despite engaging in SDM, a significant proportion of patients (50.9%) experienced decisional regret following total knee arthroplasty. This finding is consistent with other studies that have reported dissatisfaction rates ranging from 42.1% to 65% [8,9]. However, Bourne et al. (2010) [19] reported high satisfaction rates regarding pain relief (72–86%) and function for activities of daily living (70–84%). Another systematic review reported that 81% of patients were found to be ‘satisfied’ or ‘very satisfied’ following TKA [20]. These findings suggest a discrepancy in the satisfaction and regret surveys after TKA in the current literature, highlighting the need for further research to identify factors contributing to decisional regret and develop interventions to improve patient satisfaction following total knee arthroplasty.

Our study found that greater levels of DR were associated with a higher post-operative Lequesne Index and post-operative mobility status. These findings are consistent with previous research. A systematic review found that higher post-operative functional scores were the most commonly reported predictor of satisfaction, while persistent pain was the most common predictor of dissatisfaction [2]. Cassidy et al. (2021) [9] found that the improvement in Oxford Knee Score was greatest in patients without DR. This result was not surprising, since numerous studies reported that the proportion of individuals experiencing unfavorable long-term pain outcomes after knee arthroplasty ranges from 10% to 34% [21]. However, other research indicated that patients with greater preoperative pain and disability but less severe degradation in their health-related quality of life may be more satisfied after TKA [22]. These studies collectively suggested the importance of addressing patients’ emotional well-being in the preoperative and post-operative period to improve their outcomes following total knee arthroplasty.

Based on gender and age, we found no statistical differences in decisional regret scores. However, previous research has shown that women may be less satisfied with their total knee arthroplasty surgery than men, possibly due to differences in preoperative factors [3,23]. Additionally, patients aged between 70–80 have been found to be more likely to be satisfied with their surgery than those under 65 years [3]. In our study, gender and age were found to have no significance, which may be attributed to the higher average age and relatively lower socioeconomic status of our patients. Moreover, in Taiwanese clinical settings, family members are often heavily involved in surgical decisions, particularly for elderly patients. Such collectivist decision making may obscure individual-level preferences or emotional responses, thereby diluting the statistical impact of personal variables like age or sex. Additionally, the cultural emphasis on filial piety and deferring to medical authority may reduce patient self-advocacy during SDM, further limiting gender- or age-based variation. As for other influencing factors, our study found no statistical differences in the DRS scores of patients in regards to BMI, DM, ASA score, or operative time. Some studies in the literature suggest obese patients may have lower function and knee scores, but a high percentage of them are still satisfied with their knees after the operation [24]. Additionally, a prospective study including 905 Asian patients indicated that diabetic patients are no less satisfied and may even experience a greater improvement in their mental well-being and weight reduction after surgery [25]. The lack of significant differences in our study for diabetic patients may be due to our smaller sample size. Findings suggest that gender, age, BMI, DM, ASA score, and operative time might not predict patient regret after knee arthroplasty surgery.

The secondary outcomes in this article showed that post-operative mobility status and the range of motion (ROM) of the knee joint were significant predictors of higher post-operative Lequesne Index scores. Studies show post-operative mobility status and ROM are important predictors of patient satisfaction and functional outcome after knee arthroplasty [26]. Dissatisfied patients tend to have more severe functional disabilities and perceive high-flexion activities as more important. Pain associated with instability in the coronal plane and stiffness were also found to be predictors of dissatisfaction [27]. On the other hand, an increased ROM after TKA was found to be an important factor for functional outcome and satisfaction in Asian patients [28]. In addition, an increase in knee flexion, specifically exceeding 130 degrees, has been shown to have a positive impact on outcomes following total knee arthroplasty due to its influence on the fulfillment of patients’ expectations, their functional ability, and their perceptions of their knees [29]. These findings suggest that post-operative mobility status and the ROM of the knee joint should be taken into consideration in the evaluation of patients’ outcomes following knee arthroplasty surgery. Additionally, there were other factors that may also be associated with post-operative pain following TKA. An article found that preoperative anxiety was associated with pain after total knee arthroplasty, although the negative effects did not persist after a year [30]. It was also suggested that pain catastrophizing should be considered in the preoperative consultation process, as it has been shown to contribute to dissatisfaction and poor outcomes following arthroplasty [31,32,33]. Lastly, there was a study developing new prediction tools for patient satisfaction following TKA [34]. In this research, dissatisfied patients had more preoperative symptoms, such as stiffness, less pain, and a lower quality of life. However, that predictive model was based solely on preoperative parameters. Future research should focus on both preoperative and post-operative patient-reported factors, allowing surgeons and patients to better evaluate the risks and benefits of TKA surgery.

This study found that more than half of the patients undergoing primary TKAs experienced regret even following SDM counseling, highlighting the need for further research to identify factors associated with decisional regret and develop interventions to improve patient satisfaction following total knee arthroplasty. Current evidence suggests that better SDM before total joint arthroplasty may be associated with better PROMs and greater patient satisfaction 12 months later, which is an intriguing but preliminary finding [13]. Moreover, a study has indicated that patients who participated in the SDM model had higher self-reported satisfaction scores [35]. These results suggest that SDM may increase patient satisfaction. However, in our clinical practice, a relatively high level of dissatisfaction remained, even following SDM counseling. Compared to previous studies that reported increased satisfaction and PROMs with SDM, our findings diverge in that regret levels remained high despite structured SDM. This may reflect a mismatch between Western SDM models and local patient understanding. In contrast to studies conducted in Western contexts, where patients actively engage in value clarification, patients in our cohort may have engaged more passively, leading to unmet expectations despite formal SDM procedures. We could not fully determine the impact of SDM on regret because we lacked a control group. Compared to current clinical evidence, the high regret levels in our study may be caused by the older age and lower economic and educational levels of our patients, which could hinder effective communication and SDM. The patients may have had an inadequate understanding of possible post-operative symptoms, leading to unrealistic expectations about surgical efficacy. Furthermore, our study found that the primary predictors of dissatisfaction were their functional outcomes and post-operative pain, rather than preoperative features. This points out the importance of clinical efforts to improve post-operative pain management and encourage early active rehabilitation. From a theoretical perspective, anticipated regret—a forward-looking emotion predicting how one might feel if a decision leads to a poor outcome—has been widely studied in psychology and behavioral economics. In health contexts, it can heavily influence choices by shifting preferences toward lower-risk or more familiar options to avoid future regret [36]. In elective surgeries like TKA, incorporating discussions of anticipated regret into SDM can help align patient expectations with realistic outcomes. Studies have shown that prompting patients to consider potential sources of regret—such as unresolved pain or limited mobility—supports more informed and value-congruent decisions and may reduce later regret [37]. Rather than viewing regret solely as a post-decision reaction, SDM frameworks should treat it as a proactive cognitive-emotional element. Integrating anticipatory regret considerations may further enhance patient autonomy and decision satisfaction. Therefore, this study raises the issue that future SDM discussions may need to focus on comprehensively informing patients about the adverse effects of surgery and addressing overly negative expectations related to post-operative outcomes, especially in patients prone to pain catastrophizing.

This study has several strengths, including an acceptable sample size of 118 individuals and no loss to follow-up at the 3-month mark. More importantly, our findings highlight the primary reasons behind decisional regret in TKA patients following SDM counseling. The regret is largely attributed to post-operative pain and functional outcomes, suggesting that current SDM practices may not sufficiently address post-operative expectations and pain control. These results underscore a potential gap in the healthcare system regarding patient–provider interactions and the need for enhanced communication strategies.

This study has several limitations. First, it was conducted at a single center with a relatively small sample size, which may limit the generalizability of the findings. Second, the follow-up period was limited to three months, which may not fully capture the trajectory of decisional regret or long-term satisfaction after TKA. Future studies with extended follow-up periods (e.g., 6 or 12 months) are needed to assess the persistence or resolution of regret over time. Third, the absence of a non-SDM control group prevents direct comparisons of the effectiveness of shared decision making for reducing regret. A randomized controlled design including a conventional care group would provide more robust evidence for the specific impact of SDM. Finally, although the SDM process in this study included structured interviews and educational materials, the overall counseling duration was relatively brief. This may have limited the depth of patient understanding and their ability to fully integrate their preferences into the decision process. Furthermore, the study’s Taiwanese cohort has unique cultural attitudes toward surgery and medical decision making. In Taiwan, patients often defer to physician authority, and decision making is frequently guided by family consensus rather than individual autonomy. This may result in patients agreeing to surgery without fully internalizing the implications of their choices, which could explain the persistence of regret even after SDM. These cultural dynamics limit the generalizability of our findings to more individualistic healthcare systems.

In the future, there will likely be an increased emphasis on post-operative pain control and rehabilitation for patients who have undergone TKA and engaged in SDM. Additionally, future SDM discussions should shed light on the potential adverse effects of TKA to better manage patient expectations and reduce the likelihood of high post-operative expectations, which can lead to decisional regret. This may involve implementing various interventions such as early mobilization, physical therapy, and multimodal pain management strategies. By focusing on these aspects, healthcare providers can improve patient outcomes, reduce the risk of decisional regret, and enhance the overall quality of care provided to patients undergoing knee arthroplasty surgery.

## 5. Conclusions

The study found that more than half of the patients undergoing primary TKA experienced regret even following SDM counseling. Regret levels were associated with higher post-operative pain and lower mobility. This underscores the importance of informing patients about the potential adverse effects of TKA to manage their expectations and reduce regret in future SDM interviews.

### Practice Implications

This study incorporated patient perspectives through their direct engagement in the SDM process prior to surgery. Patients participated in the design of the SDM framework, which included educational pamphlets and structured interviews to assess their values and preferences. Their involvement ensured that the SDM procedure was tailored to patient-centered outcomes. Furthermore, the follow-up assessments were conducted with patients to evaluate decisional regret and post-operative outcomes, providing valuable insights into the effectiveness of the SDM process. By actively participating in the research through decision making and outcome reflection, the patients contributed to our understanding of the factors influencing decisional regret in those undergoing TKA.

## Figures and Tables

**Table 1:** Patient characteristics.

	Mean (SD) or *n* (%)
Decision regret score	18.22 (24.69)
Sociodemographic	
Gender	
Female	87 (73.7%)
Male	31 (26.3%)
Age	72.31 (7.83)
BMI	28.16 (4.82)
CCI	0.64 (0.99)
Diabetes	
Yes	23 (19.5%)
No	95 (80.5%)
Smoking	
Yes	6 (5.1%)
No	112 (94.9%)
Level of education	
Primary school or below	65 (55.0%)
High school	37 (31.4%)
College or above	16 (13.6%)
Side of TKR	
Right	67 (56.8%)
Left	51 (43.2%)
Previous history of contralateral TKR	
Yes	22 (18.6%)
No	96 (81.4%)
Preoperative Variables	
ASA grade	2.15 (0.38)
Outpatient visits within 12 months prior to surgery	3.58 (2.99)
Preoperative Lequesne Index	12.25 (3.04)
Duration of symptoms present prior to surgery (year)	4.64 (3.80)
Surgical Variables	
Operation time (min)	134.58 (28.69)
Blood loss (mL)	37.97 (61.72)
Post-operative Variables	
Mobility status	
Walks freely	97 (82.2%)
Walker	19 (16.1%)
Bedridden	2 (1.7%)
ROM of knee joint (degree)	
>90	107 (90.7%)
<90	11 (9.3%)
Post-operative 3-month Lequesne Index	5.02 (4.68)
Length of stay (day)	6.05 (1.80)

**Table 2:** Primary outcome of decision regret scale.

	No Regret	Mild Regret	Moderate-to-Severe Regret	Total	*p*-Value
Sample size (%)	58 (49.1)	29 (24.6)	31 (26.3)	118 (100)	
Average (SD) of DRS	0.00 (0.00)	17.07 (8.08)	53.39 (19.89)	18.22 (24.69)	
Sociodemographic					
Gender					0.469
Female (%)	40	22	25	87	
Male (%)	18	7	6	31	
Age (SD)	73.05 (7.91)	71.72 (8.10)	71.48 (7.55)	72.31 (7.83)	0.602
BMI (SD)	28.14 (4.35)	27.71 (3.80)	28.63 (6.38)	28.16 (4.82)	0.763
CCI (SD)	0.64 (1.14)	0.59 (0.83)	0.68 (0.87)	0.64 (0.99)	0.939
Diabetes					0.105
Yes (%)	8	5	10	23	
No (%)	50	24	21	95	
Smoking					0.726
Yes (%)	2	2	2	6	
No (%)	56	27	29	112	
Level of education					0.427
Primary school or below (%)	36	13	16	65	
High school (%)	15	10	12	37	
College or above (%)	7	6	3	16	
Side of TKR					0.957
Right (%)	33	17	17	67	
Left (%)	25	12	14	51	
Previous history of contralateral TKR					0.118
Yes (%)	14	6	2	22	
No (%)	44	23	29	96	
Preoperative Variables					
ASA grade (SD)	2.10 (0.36)	2.28 (0.46)	2.13 (0.34)	2.15 (0.38)	0.132
Outpatient visits within 12 months prior to surgery (SD)	3.76 (3.28)	3.52 (2.54)	3.32 (2.89)	3.58 (2.99)	0.802
Preoperative Lequesne Index (SD)	12.19 (3.26)	12.41 (2.69)	12.19 (3.02)	12.25 (3.04)	0.944
Duration of symptoms present prior to surgery (year) (SD)	4.35 (3.25)	4.83 (4.45)	5.01 (4.19)	4.64 (3.80)	0.704
Surgical Variables					
Operation time (min) (SD)	137.59 (30.85)	130.76 (25.72)	132.52 (27.33)	134.58 (28.69)	0.523
Blood loss (mL) (SD)	37.93 (6.26)	38.28 (48.11)	37.74 (73.02)	37.97 (61.72)	0.999
Post-operative Variables					
Mobility status					0.005
Independent mobility (Walks freely) (%)	54	24	19	97	
Limited mobility (walker) (%)	3	5	11	19	
Dependent mobility (bedridden) (%)	1	0	1	2	
ROM of knee joint					0.311
>90 (%)	55	25	27	107	
<90 (%)	3	4	4	11	
Post-operative at 3 months Lequesne Index (SD)	3.64 (3.35)	4.59 (3.75)	8.00 (6.17)	5.02 (4.68)	0.000
Length of stay (SD)	5.95 (1.67)	6.48 (2.44)	5.84 (1.21)	6.05 (1.80)	0.320

**Table 3:** Secondary outcome of post-operative Lequesne Index.

	Post-Operative Lequesne Index (SD) (CI Interval)	Total	*p*-Value (CI Interval)	Unstandardized Coefficient
Average	5.02 (4.68)	118		
Sociodemographic				
Gender			0.238 (−3.097~0.776)	
Female	5.32 (5.05)	87		
Male	4.16 (3.40)	31		
Age		72.31	0.668	0.024
BMI		28.16	0.171	0.123
CCI		0.64	0.068	0.795
Diabetes			0.013 (−4.787~−0.571)	
Yes	7.17 (6.44)	23		
No	4.49 (4.02)	95		
Smoking			0.368 (−2.116~5.664)	
Yes	3.33 (2.66)	6		
No	5.11 (4.76)	112		
Level of education			0.413	
Primary school or below	5.26 (4.97) (4.03~6.49)	65		
High school	5.22 (4.38) (3.76~6.68)	37		
College or above	3.56 (4.13) (1.36~5.76)	16		
Side of TKR			0.902 (−1.839~1.623)	
Right	4.97 (4.95)	67		
Left	5.08 (4.36)	51		
Previous history of contralateral TKR			0.985 (−2.181~2.223)	
Yes	5.00 (3.74)	22		
No	5.02 (4.89)	96		
Preoperative Variables				
ASA grade		2.15	0.289	1.199
Outpatient visits within 12 months prior to surgery		3.58	0.224	−0.177
Preoperative Lequesne Index		12.25	0.005	0.394
Duration of symptoms present prior to surgery (year)		4.64	0.767	0.034
Surgical Variables				
Operation time (min)		134.58	0.519	−0.01
Blood loss (mL)		37.97	0.163	−0.01
Post-operative Variables				
Mobility status			0.000	
Independent mobility (walks freely)	3.98 (3.56)(3.26~4.70)	97		
Limited mobility (walker)	9.58 (5.72)(6.82~12.34)	19		
Dependent mobility (bedridden)	12.00 (12.73)(−102~126)	2		
ROM of knee joint			0.002 (−7.32~−1.66)	
>90	4.60 (4.37)	107		
<90	9.09 (5.86)	11		
Length of stay		6.05	0.151	0.347

**Table 4:** Multivariate linear regression coefficients for predicting post-operative Lequesne Index.

Independent Variables	*n* (%)	B	SE B	β	*p*-Value
Constant		0.559	1.532		0.716
Diabetes	23(19.5)	1.638	0.953	0.139	0.088
No diabetes	95(80.5)		Reference group		
Preop. Lequesne Index		0.247	0.124	0.160	0.050
Mobility status (bedridden)	2(1.7)	4.077	3.162	0.113	0.200
Mobility status (walker)	19(16.1)	4.874	1.021	0.384	0.000
Mobility status (walks freely)	97(82.2)		Reference group		
ROM of knee joint (<90 degree)	11(9.3)	2.846	1.403	0.177	0.045
ROM of knee joint (>90 degree)	107(90.7)		Reference group		

## Data Availability

All data are publicly available.

## Supplementary Materials

The following supporting information can be downloaded at: https://www.mdpi.com/article/10.3390/healthcare13131597/s1. Table S1. Items of the Decision Regret Scale (DRS).

## References

[1] Scott C., Howie C., MacDonald D., Biant L. (2010). Predicting dissatisfaction following total knee replacement: A prospective study of 1217 patients. J. Bone Jt. Surg. Br. Vol..

[2] Kahlenberg C.A., Nwachukwu B.U., McLawhorn A.S., Cross M.B., Cornell C.N., Padgett D.E. (2018). Patient satisfaction after total knee replacement: A systematic review. HSS J..

[3] Baker P., Van der Meulen J., Lewsey J., Gregg P. (2007). The role of pain and function in determining patient satisfaction after total knee replacement: Data from the National Joint Registry for England and Wales. J. Bone Jt. Surg. Br. Vol..

[4] Dailiana Z.H., Papakostidou I., Varitimidis S., Liaropoulos L., Zintzaras E., Karachalios T., Michelinakis E., Malizos K.N. (2015). Patient-reported quality of life after primary major joint arthroplasty: A prospective comparison of hip and knee arthroplasty. BMC Musculoskelet. Disord..

[5] Dhurve K., Scholes C., El-Tawil S., Shaikh A., Weng L.K., Levin K., Fritsch B., Parker D., Coolican M. (2017). Multifactorial analysis of dissatisfaction after primary total knee replacement. Knee.

[6] Jacobs C.A., Christensen C.P. (2014). Factors influencing patient satisfaction two to five years after primary total knee arthroplasty. J. Arthroplast..

[7] Ali A., Lindstrand A., Sundberg M., Flivik G. (2017). Preoperative anxiety and depression correlate with dissatisfaction after total knee arthroplasty: A prospective longitudinal cohort study of 186 patients, with 4-year follow-up. J. Arthroplast..

[8] Zabawa L., Li K., Chmell S. (2019). Patient dissatisfaction following total knee arthroplasty: External validation of a new prediction model. Eur. J. Orthop. Surg. Traumatol..

[9] Cassidy R.S., Bennett D.B., Beverland D.E., O’Brien S. (2021). Decision regret after primary hip and knee replacement surgery. J. Orthop. Sci..

[10] Shimanoff S.B. (1984). Commonly named emotions in everyday conversations. Percept. Mot. Ski..

[11] Becerra Pérez M.M., Menear M., Brehaut J.C., Legare F. (2016). Extent and predictors of decision regret about health care decisions: A systematic review. Med. Decis. Mak..

[12] Faiman B., Tariman J.D. (2019). Shared Decision Making: Improving Patient Outcomes by Understanding the Benefits of and Barriers to Effective Communication. Clin. J. Oncol. Nurs..

[13] Chrenka E.A., Solberg L.I., Asche S.E., Dehmer S.P., Ziegenfuss J.Y., Whitebird R.R., Norton C.K.M., Reams M.M., Johnson P.G., Elwyn G.M.B. (2022). Is shared decision-making associated with better patient-reported outcomes? a longitudinal study of patients undergoing total joint arthroplasty. Clin. Orthop. Relat. Res..

[14] Suarez-Almazor M.E., Richardson M., Kroll T.L., Sharf B.F. (2010). A qualitative analysis of decision-making for total knee replacement in patients with osteoarthritis. JCR J. Clin. Rheumatol..

[15] Torrente-Jimenez R.S., Feijoo-Cid M., Rivero-Santana A.J., Perestelo-Pérez L., Torres-Castaño A., Ramos-García V., Bilbao A., Serrano-Aguilar P. (2022). Gender differences in the decision-making process for undergoing total knee replacement. Patient Educ. Couns..

[16] Agency for Healthcare Research and Quality (2020). The SHARE Approach—Essential Steps of Shared Decisionmaking: Quick Reference Guide.

[17] Brehaut J.C., O’Connor A.M., Wood T.J., Hack T.F., Siminoff L., Gordon E., Feldman-Stewart D. (2003). Validation of a decision regret scale. Med. Decis. Mak..

[18] Ghidini F., Sekulovic S., Castagnetti M. (2016). Parental decisional regret after primary distal hypospadias repair: Family and surgery variables, and repair outcomes. J. Urol..

[19] Bourne R.B., Chesworth B.M., Davis A.M., Mahomed N.N., Charron K.D. (2010). Patient satisfaction after total knee arthroplasty: Who is satisfied and who is not?. Clin. Orthop. Relat. Res..

[20] Gustke K., Golladay G., Roche M., Jerry G., Elson L., Anderson C. (2014). Increased satisfaction after total knee replacement using sensor-guided technology. Bone Jt. J..

[21] Beswick A.D., Wylde V., Gooberman-Hill R., Blom A., Dieppe P. (2012). What proportion of patients report long-term pain after total hip or knee replacement for osteoarthritis? A systematic review of prospective studies in unselected patients. BMJ Open.

[22] Maratt J.D., Lee Y.-y., Lyman S., Westrich G.H. (2015). Predictors of satisfaction following total knee arthroplasty. J. Arthroplast..

[23] Mehta S., Perruccio A., Palaganas M., Davis A. (2015). Do women have poorer outcomes following total knee replacement?. Osteoarthr. Cartil..

[24] Krushell R.J., Fingeroth R.J. (2007). Primary total knee arthroplasty in morbidly obese patients: A 5-to 14-year follow-up study. J. Arthroplast..

[25] Teo B.J., Chong H.-C., Yeo W., Tan A.H. (2018). The impact of diabetes on patient outcomes after total knee arthroplasty in an Asian population. J. Arthroplast..

[26] Kim T.K., Kwon S.K., Kang Y.G., Chang C.B., Seong S.C. (2010). Functional disabilities and satisfaction after total knee arthroplasty in female Asian patients. J. Arthroplast..

[27] Howells N., Murray J., Wylde V., Dieppe P., Blom A. (2016). Persistent pain after knee replacement: Do factors associated with pain vary with degree of patient dissatisfaction?. Osteoarthr. Cartil..

[28] Ha C.-W., Park Y.-B., Song Y.-S., Kim J.-H., Park Y.-G. (2016). Increased range of motion is important for functional outcome and satisfaction after total knee arthroplasty in Asian patients. J. Arthroplast..

[29] Devers B.N., Conditt M.A., Jamieson M.L., Driscoll M.D., Noble P.C., Parsley B.S. (2011). Does greater knee flexion increase patient function and satisfaction after total knee arthroplasty?. J. Arthroplast..

[30] Wylde V., Trela-Larsen L., Whitehouse M.R., Blom A.W. (2017). Preoperative psychosocial risk factors for poor outcomes at 1 and 5 years after total knee replacement: A cohort study of 266 patients. Acta Orthop..

[31] Vissers M.M., Bussmann J.B., Verhaar J.A., Busschbach J.J., Bierma-Zeinstra S.M., Reijman M. (2012). Psychological factors affecting the outcome of total hip and knee arthroplasty: A systematic review. Seminars in Arthritis and Rheumatism.

[32] Burns L.C., Ritvo S.E., Ferguson M.K., Clarke H., Seltzer Ze Katz J. (2015). Pain catastrophizing as a risk factor for chronic pain after total knee arthroplasty: A systematic review. J. Pain Res..

[33] Lewis G., Rice D., McNair P., Kluger M. (2015). Predictors of persistent pain after total knee arthroplasty: A systematic review and meta-analysis. Br. J. Anaesth..

[34] Van Onsem S., Van Der Straeten C., Arnout N., Deprez P., Van Damme G., Victor J. (2016). A new prediction model for patient satisfaction after total knee arthroplasty. J. Arthroplast..

[35] Stacey D., Hawker G., Dervin G., Tugwell P., Boland L., Pomey M.-P., O’cOnnor A.M., Taljaard M. (2014). Decision aid for patients considering total knee arthroplasty with preference report for surgeons: A pilot randomized controlled trial. BMC Musculoskelet. Disord..

[36] Zeelenberg M., Pieters R. (2007). A theory of regret regulation 1.0. J. Consum. Psychol..

[37] Bekker H.L., Winterbottom A.E., Butow P., Dillard A.J., Feldman-Stewart D., Fowler F.J., Jibaja-Weiss M.L., A Shaffer V., Volk R.J. (2013). Do personal stories make patient decision aids more effective? A critical review of theory and evidence. BMC Med Inf. Decis Mak.

