# Supplementary material for: Factors Associated with Decisional Regret After Shared Decision Making for Patients Undergoing Total Knee Arthroplasty
Source: Healthcare (Basel). 2025 Jul 3;13(13):1597. doi: 10.3390/healthcare13131597 (PMC12250144; doi:10.3390/healthcare13131597)
Supplement: Supplementary file 1 [file healthcare-13-01597-s001.zip › healthcare-3679730-supplementary.pdf]

Supplementary Table S1. Items of the Decision Regret Scale (DRS)

**Item**	**1 Strongly Agree**	**2 Agree**	**3 Neither Agree nor Disagree**	**4 Disagree**	**5 Strongly Disagree**
1. It was the right decision.	☐	☐	☐	☐	☐
2. I regret the choice that was made. *(reverse scored)*	☐	☐	☐	☐	☐
3. I would go for the same choice if I had to do it over again.	☐	☐	☐	☐	☐
4. The choice did me a lot of harm. *(reverse scored)*	☐	☐	☐	☐	☐
5. The decision was a wise one.	☐	☐	☐	☐	☐
Scoring: Items 2 and 4 are reverse scored. All items are summed, converted to a 0–100 scale, with higher scores indicating greater regret.					
